# Longitudinal health-related quality of life in people with thoracic aortic aneurysms

**DOI:** 10.1093/bjs/znae228

**Published:** 2024-09-11

**Authors:** Linda D Sharples, Vasiliki Anagnostopoulou, Anna L Pouncey, Carol Freeman, Andrew McCarthy, Joanne Gray, Peter McMeekin, Priya Sastry, Luke Vale, Colin Bicknell, Stephen R Large

**Affiliations:** Department of Medical Statistics, London School of Hygiene and Tropical Medicine, London, UK; Department of Medical Statistics, London School of Hygiene and Tropical Medicine, London, UK; Department of Surgery and Cancer, Imperial College London, St Mary’s Hospital, London, UK; Papworth Department of Surgery and Trials Unit Collaboration, Royal Papworth Hospital NHS Foundation Trust, Cambridge, UK; Faculty of Health and Life Sciences, Northumbria University, Newcastle upon Tyne, UK; Faculty of Health and Life Sciences, Northumbria University, Newcastle upon Tyne, UK; Faculty of Health and Life Sciences, Northumbria University, Newcastle upon Tyne, UK; Department of Cardiac Surgery, John Radcliffe Hospital, Oxford, UK; Department of Medical Statistics, London School of Hygiene and Tropical Medicine, London, UK; Department of Surgery and Cancer, Imperial College London, St Mary’s Hospital, London, UK; Papworth Department of Surgery and Trials Unit Collaboration, Royal Papworth Hospital NHS Foundation Trust, Cambridge, UK

## Abstract

**Background:**

Surgical intervention for thoracic aortic aneurysms is high risk. Understanding changes in health-related quality of life before and after endovascular stent grafting and open surgical repair can aid treatment decision-making.

**Methods:**

The Effective Treatments for Thoracic Aortic Aneurysms (‘ETTAA’) study (ISRCTN04044627) was a longitudinal, observational study. Adults with new/existing arch or descending thoracic aortic aneurysms greater than or equal to 4 cm in diameter were followed from 2014 to 2022. Five domains of health-related quality of life (Mobility, Self-Care, Usual Activities, Pain/Discomfort, and Anxiety/Depression) were recorded using the EuroQoL, five dimensions, five levels (‘EQ-5D-5L’) questionnaire and analysed using a range of longitudinal mixed models.

**Results:**

Of 886 thoracic aortic aneurysm participants, 824 completed at least 2 questionnaires. Patients had slightly worse health-related quality of life than age-matched norms. Without surgery, deterioration occurred over time in Mobility (0.072/year (95% c.i. 0.042 to 0.101), *P* < 0.001) and Self-Care (0.039/year (95% c.i. 0.018 to 0.061), *P* < 0.001) in both sexes and Pain/Discomfort in women (0.069/year (95% c.i. 0.020 to 0.118), *P* = 0.005). For 6 weeks after endovascular stent grafting, there was a significant impairment in Self-Care (0.214 (95% c.i. 0.112 to 0.316), *P* < 0.001) and (for women only) in Usual Activities (0.625 (95% c.i. 0.338 to 0.911), *P* < 0.001), which then returned to pre-endovascular stent grafting levels. Six weeks after open surgical repair, the impairment in health-related quality of life was greater (Mobility 0.492 (95% c.i. 0.314 to 0.669), Self-Care 0.474 (95% c.i. 0.364 to 0.583), Usual Activities 1.469 (95% c.i. 1.042 to 1.896), and Pain/Discomfort 0.561 (95% c.i. 0.363 to 0.760), all *P* < 0.001) and took longer to return to pre-open surgical repair levels, partly due to increased complications and longer hospitalization. Anxiety/Depression decreased after open surgical repair (−0.214 (95% c.i. −0.326 to −0.101), *P* < 0.001). Age, sex, frailty, smoking, New York Heart Association class, and chronic obstructive pulmonary disease were significantly associated with health-related quality of life.

**Conclusion:**

Without intervention, health-related quality of life declines as age increases. Changes in health-related quality of life should contribute to surgical treatment decision-making.

## Teaser

Understanding changes in health-related quality of life before and after endovascular stent grafting and open surgical repair can aid treatment decision-making. For 6 weeks after endovascular stent grafting, there was a significant impairment in Self-Care (0.214 (95% c.i. 0.112 to 0.316), *P* < 0.001) and (for women only) in Usual Activities (0.625 (95% c.i. 0.338 to 0.911), *P* < 0.001), which then returned to pre-endovascular stent grafting levels. Six weeks after open surgical repair, the impairment in health-related quality of life was greater (Mobility 0.492 (95% c.i. 0.314 to 0.669), Self-Care 0.474 (95% c.i. 0.364 to 0.583), Usual Activities 1.469 (95% c.i. 1.042 to 1.896), and Pain/Discomfort 0.561 (95% c.i. 0.363 to 0.760), all *P* < 0.001) and took longer to return to pre-open surgical repair levels. Anxiety/Depression decreased after open surgical repair (−0.214 (95% c.i. −0.326 to −0.101), *P* < 0.001).

## Introduction

To understand the benefit of chronic thoracic aortic aneurysm (CTAA) repair to patients and to aid shared decision-making for patients and clinicians, information on health-related quality of life (HRQoL), as well as the risk of non-fatal events and mortality, both before and after repair, is needed^1–4^. Published evidence is inconclusive and does not clearly correlate with biomedical symptoms^1–3^. A 2019 review of studies of HRQoL after thoracic aortic surgery called for more information on HRQoL surrounding cardiac surgery in general, including time taken to recover after surgery and ability to return to ‘living life to the fullest’^4^.

The Effective Treatments for Thoracic Aortic Aneurysms (ETTAA) study was a prospective 5-year observational study that recruited 886 participants with CTAA. As part of the ETTAA study, an evaluation of aneurysm growth, survival, clinical events, and hospital admissions without surgical intervention was published, as was micro-costing of endovascular stent grafting (ESG) and open surgical repair (OSR) within the UK National Health Service (NHS) setting^5,6^. Further details of the study and its findings are published elsewhere^7^.

A secondary objective of the ETTAA study was to assess HRQoL, measured via the EuroQoL, five dimensions, five levels (EQ-5D-5L) questionnaire. Responses to the questions in the EQ-5D-5L questionnaire can be combined into a single measure of utility, with one representing maximum possible health and zero representing death^8,9^. Previous analysis concentrated on the EQ-5D-5L questionnaire utility value and analysed pre-intervention and post-intervention intervals separately^7,10^. Baseline utility scores were slightly lower than the age-adjusted UK population norms^11^. The main finding was that pre-intervention EQ-5D-5L questionnaire utility varied at baseline, which could be partially explained by age, sex, current smoking, and New York Heart Association (NYHA) class. Decline in HRQoL was faster for older participants and current smokers, but there was substantial unexplained variation between participants in the rate of change over time. Post-intervention, there was a small decrease in utility during the first 6 weeks post-ESG, but no evidence that utility changed over time thereafter. There was a larger decrease in the first 6 weeks post-OSR. After these initial 6 weeks, there was evidence of a difference in utility between ESG and OSR for participants who survived.

Although EQ-5D-5L questionnaire utility values are useful for economic evaluations, to understand how quality of life changes whilst patients are monitored before surgery and for patients after ESG and OSR, each EQ-5D-5L questionnaire domain must be examined. Analysis may help to improve the treatment of people with CTAA by providing clinicians and patients with additional information on how patient characteristics, waiting time, and subsequent treatment affect HRQoL.

## Methods

### Declaration of Helsinki

The authors confirm that the ETTAA study complies with the Declaration of Helsinki, that the West Midlands—South Birmingham Research Ethics Committee approved the research protocol, and that informed consent was obtained from all participants.

### Participants

The ETTAA study recruited 886 participants with CTAA from 30 English NHS centres between 24 March 2014 and 24 July 2018. Patients were included when aged greater than or equal to 18 years and when they presented to NHS hospitals with existing or new aneurysms in the arch or descending aorta of greater than or equal to 4 cm in diameter, without previous intervention for the same aneurysm or acute dissection.

Recruitment was prompted by diagnosis of CTAA or referral for consideration of intervention, so that stage of disease at baseline varied between participants. At recruitment, participants were categorized according to intended treatment: conservative management (CM) for patients who were not intended to have any surgical procedures due to patient choice, co-morbidities, or surgical risks; ESG; OSR; or watchful waiting (WW) for patients with smaller aneurysms at low risk of rupture, who were not referred for surgery, but for whom surgery could be a future option.

Some participants moved between groups during the ETTAA study; the final analysis and descriptive statistics are based on the numbers of participants in each group at the end of the study.

### Health-related quality of life

The EQ-5D-5L questionnaire records five domains of HRQoL (Mobility, Self-Care, Usual Activities, Pain/Discomfort, and Anxiety/Depression), each on a five-point Likert scale, from one (no problems) to five (severe impairment). Participants were followed up between 1 and 5 years after referral and scheduled to complete questionnaires at consent and 3, 6, 12, 18, 24, 36, and 48 months before having an intervention. When surgery took place, questionnaire completion was rescheduled at 1, 3, 6, 12, 18, 24, 36, and 48 months thereafter.

### Statistical methods

See the *Supplementary material* for details. Longitudinal data were analysed as repeated measures in continuous time starting from the baseline questionnaire.

Linear mixed effects models were used, assuming that the five levels of each domain represent a latent continuous variable that describes participants’ HRQoL. This allows easy interpretation of the coefficients. Time, time-squared (for non-linear relationships), participant baseline characteristics (age, height, BMI, sex, social care, and smoking history), aneurysm features (maximum diameter and location in aorta), co-morbidities (extracardiac arteriopathy, heart valve disease, connective tissue disorder (CTD), coronary artery disease, chronic obstructive pulmonary disease (COPD), and NYHA class), and treatment with statins were considered as fixed effects. Treatment group/interval was also considered (CM, ESG pre-intervention, ESG within 6 weeks, ESG greater than 6 weeks post-intervention, OSR pre-intervention, OSR within 6 weeks, OSR greater than 6 weeks after intervention, and WW).

Random effects were included for intercept and slope to model additional variability in HRQoL between individuals at recruitment and over time. Different variance for the NYHA classes was incorporated. Final models were decided using Wald tests and Akaike Information Criteria (AIC)/Bayesian Information Criteria (BIC). Postoperative outcomes and complications during admission were summarized and relationships with HRQoL explored using logistic and linear regression.

### Missing data and sensitivity analysis

Different numbers of forms were completed for each participant because study entry, duration of follow-up, and incidence of intervention/re-intervention (with intervention prompting further data collection) varied. Detailed analysis (see the *Supplementary material*) found little to no evidence against an assumption of ‘missing completely at random’. Therefore, complete-case analysis is presented.

Sensitivity of results to missing data was assessed by multiple imputation using chained equations^12^. Joint models of HRQoL and survival were fitted to assess sensitivity due to those with the worst HRQoL dropping out due to ill health (see the *Supplementary material*)^13^.

Sensitivity of linear model results to using bootstrapped standard errors and generalized estimating equations with robust standard errors was assessed. Mixed effects proportional odds and probit models were also fitted for each domain (see the *Supplementary material*).

## Results

### Descriptive statistics

**Fig. 1:**
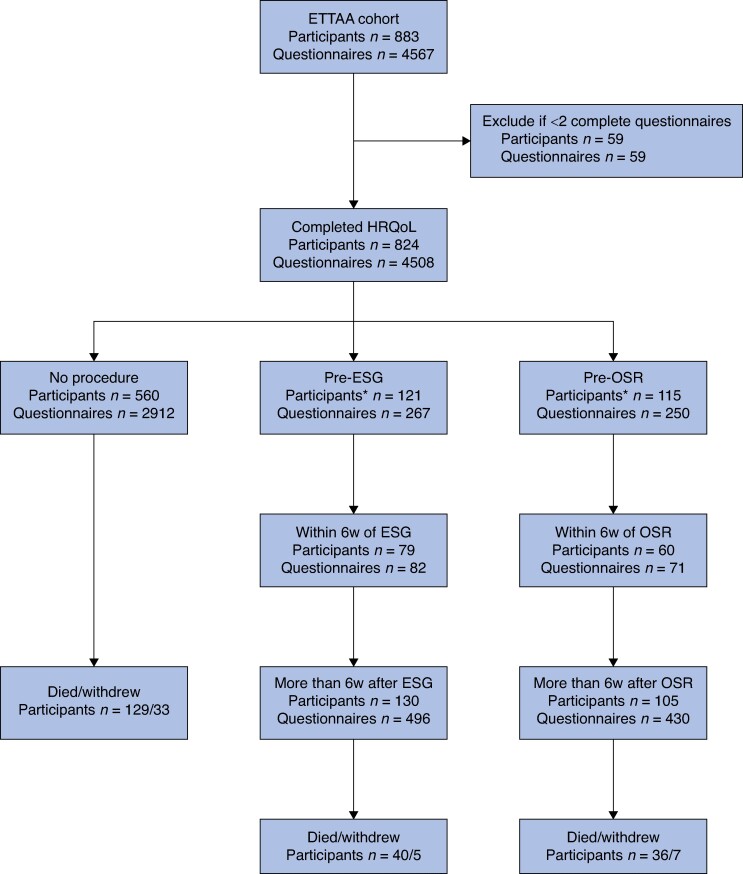
Flow chart showing number of participants and EuroQoL, five dimensions, five levels (‘EQ-5D-5L’) questionnaires completed during the Effective Treatments for Thoracic Aortic Aneurysms study *Twenty-three endovascular stent grafting participants and five open surgical repair participants only completed questionnaires after their procedures. ETTAA, Effective Treatments for Thoracic Aortic Aneurysms; HRQoL, health-related quality of life; ESG, endovascular stent grafting; OSR, open surgical repair; 6w, 6 weeks.

A total of 886 participants were recruited to the ETTAA study between 24 March 2014 and 24 July 2018, and followed up for at least 1 year to 31 August 2019 (*Fig. 1*). By the end of the study, 205 participants (23.1%) had died and 45 participants (5.1%) had withdrawn. The mean(s.d.) follow-up time was 2.5(1.2) years (range 0.1–5.2 years) for those who survived to the end of the ETTAA study and the mean(s.d.) time to death was 1.4(1.1) years (range 0.003–4.2 years) for those who died.

Participant characteristics, co-morbidities, and use of medication at recruitment are presented in *Tables S1*, *S2* and have been reported in full elsewhere^5^. There were 321 women (36.2%) and 565 (63.8%) men. The mean(s.d) age at baseline was 70.8(10.9) years (range 26.1–92.5 years). On average, women were older than men (mean(s.d.) age of 72.8(9.9) years *versus* 69.7(11.2) years respectively). Of 321 women, 93 (29.0%) died during the study, compared with 112 of 565 (19.8%) men.

The 886 participants were classified as WW (489 participants), CM (112 participants), ESG (150 participants), and OSR (135 participants). CM participants were older and more likely to require formal/informal care than other groups. OSR participants were younger and less likely to need care than the other groups. Overall, 601 participants did not have a procedure, 246 participants (138 ESG participants and 108 OSR participants) had 1 procedure, 36 participants (12 ESG participants and 24 OSR participants) had 2 procedures, and 3 participants (all OSR participants) had 3 procedures. There were important differences between ESG and OSR participants, which may be reflected in their HRQoL^7^. ESG participants were older and had more co-morbidities. OSR participants were less likely to be smokers, suffer from COPD, or take statins, but were more likely to have CTD (14.8% *versus* 1.3% of participants) and were younger. Importantly, 100 of 135 OSR participants were not eligible for ESG, due to concomitant cardiac surgery, location of the aneurysm, or presence of CTD. Similarly, 35 of 150 ESG participants were not eligible for OSR, due to age, frailty, or operative risk. No direct comparisons of ESG and OSR were conducted^7^. Complications were common in both groups, with most being temporary, albeit with participants requiring prolonged ICU and hospital stays (*Table S3*). In the ESG group, 8 participants died during the surgical admission, five participants required prolonged ventilation (greater than 48 h), 4 participants had strokes, 9 participants had myocardial infarctions, and 5 participants had spinal cord injuries (3 participants had paraplegia and 2 participants had paraparesis). After OSR, 15 participants died in hospital, 37 participants required prolonged ventilation, 11 participants had strokes, 2 participants had myocardial infarctions, and 4 participants had paraplegia.

### EuroQoL, five dimensions, five levels (‘EQ-5D-5L’) questionnaire domains

A total of 883 participants completed 4567 (range 1–13, mean 5.17) EQ-5D-5L questionnaires (*Fig. 1*). To consider changes in HRQoL, 824 participants with at least 2 questionnaires were included. Overall, 3429 questionnaires were completed before any procedure took place, 2912 by participants who had no procedure during the ETTAA study and 513 by participants who subsequently had surgery; 1079 were completed after surgery.

Baseline scores are presented in *Table S4*. Most participants reported no problems in all five domains. Self-Care was least affected, with 396 WW participants (81.0%), 81 CM participants (72.3%), 117 ESG participants (78.0%), and 118 OSR participants (87.4%) reporting no problems at baseline. Fewer CM participants reported no problems for Mobility (32.1%) and Usual Activities (37.5%) compared with the other three groups. Approximately half the participants reported no problems in the other domains. This is lower than the age-matched UK population that reported no problems for Mobility (65.6%), Self-Care (90.1%), Usual Activities (73.1%) Pain/Discomfort (48.0%), and Anxiety/Depression (80.2%)^11^.

### Linear mixed effects models

Full models for the five domains are provided in *Tables S5–S9*.

#### Changes in health-related quality-of-life domains over time

**Fig. 2:**
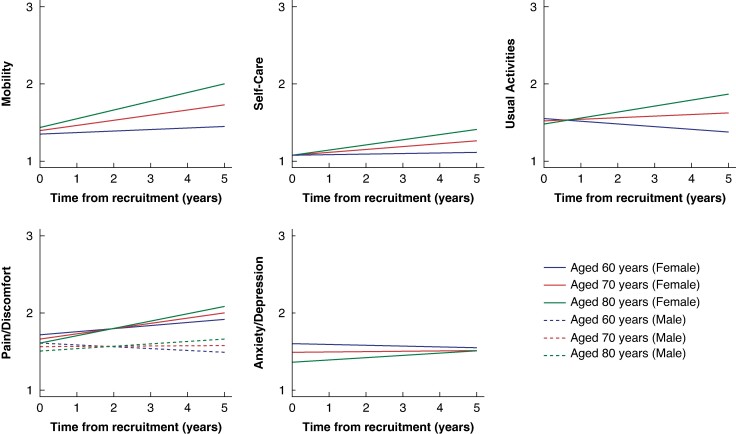
Estimated mean changes from baseline during watchful waiting for the five domains of the EuroQoL, five dimensions, five levels (‘EQ-5D-5L’) questionnaire for men and women aged 60, 70, and 80 years, who were not current smokers Only Pain/Discomfort has different trajectories for men and women. Self-Care does not differ for smokers. Anxiety/Depression trajectories are plotted for participants without chronic obstructive pulmonary disease—participants with chronic obstructive pulmonary disease have higher (worse) Anxiety/Depression throughout.

There was strong evidence that Mobility, Self-Care, and Pain/Discomfort deteriorated over time, although the annual change was small (*Table 1* and *Fig. 2*). For Pain/Discomfort the effect of time was modified by sex; there was an annual increase for women, but no increase for men. For each domain, deterioration in HRQoL was greater as age at baseline increased (*Table 1* time–age interaction and *Fig. 2*) and this effect was strong in Usual Activities and Mobility, less strong in Self-Care and Pain/Discomfort, and weakest in Anxiety/Depression.

**Table 1:** Estimated change in mean score (95% c.i.) per year for a person of mean age at baseline, and additional change in score for each 10-year increase in age at baseline, in the final models for the five domains of the EuroQoL, five dimensions, five levels (‘EQ-5D-5L’) health-related quality-of-life questionnaire

Mobility (4308 measurements, 784 participants)	Self-Care (4321 measurements, 786 participants)	Usual Activities (4308 measurements, 784 participants)	Pain/Discomfort (4308 measurements, 784 participants)	Anxiety/Depression (4299 measurements, 781 participants)
**Change in score per year for a participant of average age at baseline (Time effect)**				
0.072 (0.042,0.101), *P* < 0.001*	0.039 (0.018,0.061), *P* < 0.001*	0.027 (−0.004,0.057), *P* = 0.088‡	Women 0.069 (0.020,0.118), *P* = 0.005* Men 0.004 (−0.30,0.039), *P* = 0.814‡	0.009 (−0.014,0.033), *P* = 0.423‡
**Additional change in score per year for each decade increase in age at baseline (Time by age interaction)**				
0.047 (0.021,0.073), *P* < 0.001*	0.031 (0.012,0.050), *P* = 0.001*	0.056 (0.029,0.082), *P* < 0.001*	0.028 (0.0026,0.053), *P* = 0.031†	0.020 (−0.000,0.040), *P* = 0.054‡

Higher scores correspond to worse HRQoL; Likert scale from one (no problems) to five (severe impairment). For Self-Care, participants with missing smoking status could be included in final models and, for Anxiety/Depression, participants with missing chronic obstructive pulmonary disease status were excluded. Sex was included in models for Usual Activities and Pan/Discomfort due to interactions with operative status and time respectively. *Strong evidence. †Weak evidence. ‡No/negligible evidence.

#### Changes in health-related quality-of-life domains after endovascular stent grafting and open surgical repair

**Fig. 3:**
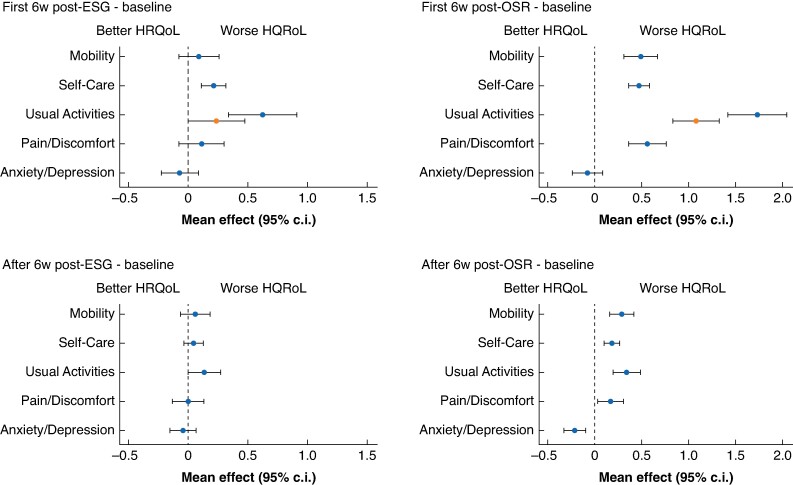
Estimated mean (95% c.i.) changes from baseline for the five domains of the EuroQoL, five dimensions, five levels (‘EQ-5D-5L’) questionnaire Top row, change in the first 6 weeks post-intervention. Bottom row, change after the first 6 weeks post-intervention. 6w, 6 weeks; ESG, endovascular stent grafting; OSR, open surgical repair; HRQoL, health-related quality of life.

Compared with before intervention, there was a small significant increase in score in the first 6 weeks after ESG in Self-Care and Usual Activities (*Table 2* and *Fig. 3*). In Usual Activities, the increase in impairment was higher for women than men. After this initial 6-week interval, scores decreased and were similar to before ESG.

**Table 2:** Estimated change in mean scores (95% c.i.) from before to after endovascular stent grafting, and from before to after open surgical repair, in the final models for the five domains of the EuroQoL, five dimensions, five levels (‘EQ-5D-5L’) health-related quality-of-life questionnaire

Mobility (4308 measurements, 784 participants)	Self-Care (4321 measurements, 786 participants)	Usual Activities (4308 measurements, 784 participants)	Pain/Discomfort (4308 measurements, 784 participants)	Anxiety/Depression (4299 measurements, 781 participants)
**Mean difference ESG within 6 weeks–ESG pre-intervention**				
0.091 (−0.078, 0.259), *P* = 0.291‡	0.214 (0.112,0.316), *P* < 0.001*	Women 0.625 (0.338,0.911), *P* < 0.001* Men 0.237 (0.000,0.474), *P* = 0.050†	0.113 (−0.075,0.301), *P* = 0.238‡	−0.071 (−0.226,0.084), *P* = 0.369‡
**Mean difference ESG after 6 weeks post-intervention–ESG pre-intervention**				
0.060 (−0.063,0.183), *P* = 0.339‡	0.046 (−0.033,0.125), *P* = 0.252‡	0.137 (−0.001,0.275), *P* = 0.051‡	0.001 (−0.131,0.134), *P* = 0.984‡	−0.042 (−0.151,0.068), *P* = 0.457‡
**Mean difference OSR within 6 weeks–OSR pre-intervention**				
0.492 (0.314,0.669), *P* < 0.001*	0.474 (0.364,0.583), *P* < 0.001*	Women 1.469 (1.042,1.896), *P* < 0.001* Men 1.081 (0.836,1.326), *P* < 0.001*	0.561 (0.363,0.760), *P* < 0.001*	−0.071 (−0.226,0.084), *P* = 0.369‡
**Mean difference OSR after 6 weeks post-intervention–OSR pre-intervention**				
0.278 (0.152,0.404), *P* < 0.001*	0.174 (0.094,0.255), *P* < 0.001*	0.328 (0.185,0.470), *P* < 0.001*	0.161 (0.024,0.297), *P* = 0.021†	−0.214 (−0.326,−0.101), *P* < 0.001*

Higher scores correspond to worse HRQoL after surgery; Likert scale from one (no problems) to five (severe impairment). For Self-Care, participants with missing smoking status could be included in final models and, for Anxiety/Depression, participants with missing chronic obstructive pulmonary disease status were excluded. *Strong evidence. †Weak evidence. ‡No/negligible evidence. ESG, endovascular stent grafting; OSR, open surgical repair.

Compared with before intervention, there was a large increase in score in the first 6 weeks after OSR in most domains, which decreased thereafter and remained significantly (but only slightly) higher than before surgery (*Table 2* and *Fig. 3*). The exception was Anxiety/Depression, which improved slightly in the first 6 weeks after OSR and was significantly better thereafter (median follow-up 2.2 years). Impairment in Usual Activities was worse for women than men.

The CM group had worse Mobility and Usual Activity scores than the WW group, with mean differences in scores of 0.318 (95% c.i. 0.149 to 0.487) and 0.306 (95% c.i. 0.134 to 0.478) respectively. Before surgery, OSR participants had better Mobility than the WW group by −0.291 (95% c.i. −0.463 to −0.119). Otherwise, HRQoL for the four management groups before any surgery was similar.

#### Additional effects of baseline variables

**Fig. 4:**
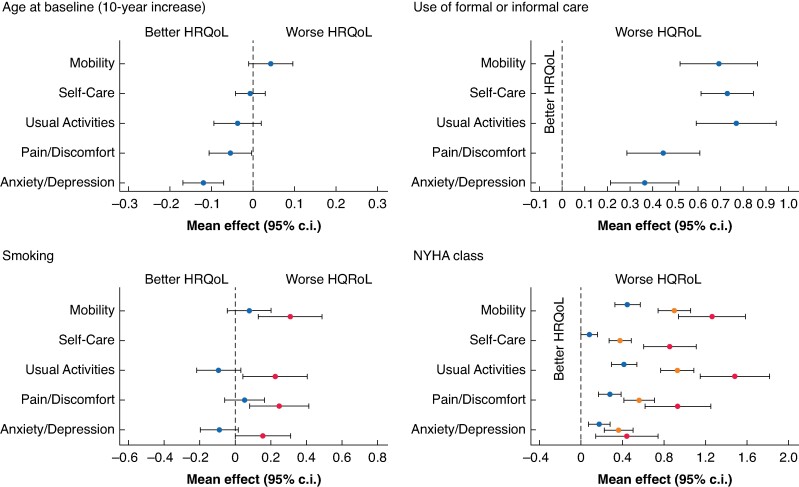
Estimated mean (95% c.i.) effect on the five domains of the EuroQoL, five dimensions, five levels (‘EQ-5D-5L’) questionnaire Top left, for each 10-year increase in age at baseline. Top right, for use of formal or informal care. Bottom left, for smoking (current smokers, and ex-smokers relative to never smokers). Bottom right, for increasing New York Heart Association classes (II, III, and IV, relative to class I). HRQoL, health-related quality of life; NYHA, New York Heart Association.

Differences in baseline HRQoL could partially be explained by age, sex, smoking, use of formal/informal social care, NYHA class, and COPD (*Table 3* and *Fig. 4*).

**Table 3:** Estimated effects on scores (95% c.i.) of baseline factors in the final models for the five domains of the EuroQoL, five dimensions, five levels (‘EQ-5D-5L’) health-related quality-of-life questionnaire

Mobility (4308 measurements, 784 participants)	Self-Care (4321 measurements, 786 participants)	Usual Activities (4308 measurements, 784 participants)	Pain/Discomfort (4308 measurements, 784 participants)	Anxiety/Depression (4299 measurements, 781 participants)
**Estimated difference in mean score for each decade increase in age at baseline**				
0.043 (−0.010,0.096), *P* = 0.112‡	−0.006 (−0.041,0.0292), *P* = 0.717‡	−0.037 (−0.094,0.020), *P* = 0.201‡	−0.054 (−0.104,−0.003), *P* = 0.038†	−0.119 (−0.017,−0.007), *P* < 0.001*
**Estimated difference in mean score for women compared to men**				
	–	0.068 (−0.048,0.184), *P* = 0.247‡	0.100 (−0.212,0.012), *P* = 0.093‡	0.142 (−0.240,−0.044), *P* = 0.004*
**Estimated difference in mean score if using formal/informal social care**				
0.693 (0.522,0.864), *P* < 0.001*	0.729 (0.613,0.844), *P* < 0.001*	0.769 (0.592,0.947), *P* < 0.001*	0.447 (0.286,0.608), *P* < 0.001*	0.366 (0.216,0.516), *P* < 0.001*
**Estimated difference in mean score for NYHA class** compared to **class I**				
II 0.446 (0.325,0.567), *P* < 0.001* III 0.899 (0.743,1.055), *P* < 0.001* IV 1.262 (0.942,1.583), *P* < 0.001*	II 0.080 (0.002,0.158), *P* = 0.046† III 0.376 (0.268,0.484), *P* < 0.001* IV 0.855 (0.602,1.108), *P* < 0.001*	II 0.415 (0.291,0.538), *P* < 0.001* III 0.929 (0.767,1.090), *P* < 0.001* IV 1.484 (1.148,1.821), *P* < 0.001*	II 0.277 (0.165,0.389), *P* < 0.001* III 0.559 (0.452,0.746), *P* < 0.001* IV 0.933 (0.615,1.251), *P* < 0.001*	II 0.174 (0.070,0.278), *P* = 0.001* III 0.361 (0.222,0.500), *P* < 0.001* IV 0.442 (0.140,0.744), *P* = 0.004*
**Estimated difference in mean score for current and past smokers compared to never smokers**				
Past 0.079 (−0.043,0.201), *P* = 0.199‡ Current 0.310 (0.132,0.488), *P* = 0.001*	–	Past −0.092 (−0.215,0.031), *P* = 0.145‡ Current 0.224 (0.041,0.407), *P* = 0.016*	Past 0.053 (−0.059,0.165), *P* = 0.356‡ Current 0.248 (0.082,0.414), *P* = 0.003*	Past −0.088 (−0.194,0.018), *P* = 0.099‡ Current 0.155 (−0.002,0.312), *P* = 0.053†
**Estimated difference in mean score for participants with chronic obstructive pulmonary disease**				
	–	–	–	0.145 (0.015,0.274), *P* = 0.028*

Higher scores correspond to worse HRQoL; Likert scale from one (no problems) to five (severe impairment). For Self-Care, participants with missing smoking status could be included in final models and, for Anxiety/Depression, participants with missing chronic obstructive pulmonary disease status were excluded. *Strong evidence. †Weak evidence. ‡No/negligible evidence. NYHA, New York Heart Association.

Participants reporting use of social care had significantly worse HRQoL in all domains, with Usual Activities most impaired and Anxiety/Depression least impaired. There was greater impairment in each domain with each increase in NYHA class, suggesting that the severity of cardiorespiratory symptoms had the biggest impact on HRQoL across all domains. This effect was particularly prominent in Usual Activities and Mobility, with the average score more than one unit higher for NYHA class IV compared with class I. There was some evidence that COPD was associated with greater Anxiety/Depression. HRQoL was worse for current smokers for all domains except Self-Care. There was strong evidence that men had less Anxiety/Depression than women.

### Missing data and sensitivity analysis

Overall, 777 participants (87.7%) had complete baseline characteristics, corresponding to 4091 questionnaires (89.5%). Refitting the final model for each domain using multiple imputation resulted in very similar estimates (example in *Table S10*). In the joint survival-longitudinal analysis, HRQoL declined to a slightly larger extent than estimated when modelling the longitudinal process alone, in all the models except for Usual Activities (example in *Tables S11*, *S12*).

All sensitivity analyses produced very similar results and conclusions, including analyses using mixed effects ordinal logistic regression (results available on request).

### Effect of operative complications on health-related quality of life

Adjusting for operation type, worse preoperative HRQoL was associated with worse postoperative HRQoL for all domains and, for OSR patients only, with having any postoperative complication (*Tables S13*, *S14*).

## Discussion

Responses to the EQ-5D-5L questionnaire in the ETTAA study revealed a slow significant decline in HRQoL across most domains, which increased with increasing age at baseline, consistent with population norms^11^. HRQoL was severely impacted by functional limitations (NYHA class) and frailty (use of formal/informal care). The only reversible factor associated with impaired HRQoL was current smoking. Participants with good preoperative HRQoL in all domains had better postoperative HRQoL. Worse preoperative HRQoL was associated with complications after OSR, but not ESG.

HRQoL declined early after ESG, for Self-Care and Usual Activities, and shifted toward pre-intervention levels thereafter. The more invasive nature of OSR meant that scores increased sharply in the first 6 weeks after intervention and remained slightly higher than before intervention thereafter. Slower recovery after OSR might be expected for open surgery, which also included concomitant cardiac procedures and vascular stenting for some participants, and resulted in high levels of postoperative complications, including stroke. The exception was Anxiety/Depression, which was similar to baseline in the first 6 weeks and decreased thereafter, suggesting that participants were less anxious or depressed after OSR. The effect of both interventions on Usual Activities was higher in the first 6 weeks for women compared with men. Women appeared to have higher Pain/Discomfort and Anxiety/Depression at baseline, and a faster increase in Pain/Discomfort over time, consistent with sex differences in HRQoL observed for both cardiovascular and other diseases^14,15^.

It is worth stressing that patients undergoing ESG and OSR differ substantially and OSR participants were often ineligible for ESG^7^. Decisions around the nature and timing of surgery for patients diagnosed with CTAA are guided by broad international guidelines^16,17^. The findings of the present study do not challenge these guidelines or favour either ESG or OSR. However, given that ESG and OSR are not without risks, patient input is important. This analysis of HRQoL can better inform participants and clinicians about likely outcomes whilst awaiting potentially life-extending surgery if the aneurysm reaches threshold size.

In a review, Gökalp and Takkenberg^4^ acknowledged the difficulties in managing patients with CTAA and recommended that patient preferences, alongside clinical evidence and patient-specific risks, should be taken into account. This requires knowledge of patient-reported HRQoL and how that might change after treatment. Importantly, clinician–patient discussions should include how the patient values different aspects of HRQoL. Development of decision aids may be an effective way to both empower patients and facilitate shared decision-making in clinical practice^18^.

Reviews of evidence on HRQoL after thoracic aortic surgery in general^1,2,19^ and hereditary TAA and dissection in particular^3^ have been published. Most studies identified in these reviews were small, were single centre, and used cross-sectional designs^20–23^. Some studies found that HRQoL after surgery was generally comparable to that of the general population, even in older patients and those presenting as emergencies, whereas others found that it was significantly lower, consistent with the findings of the present study^2,21,24–27^.

Few studies have compared HRQoL over time, or before and after surgery, so that early and longer-term impacts of intervention remain unclear. The present study addresses this gap; it found that participants in the CM group had significantly poorer EQ-5D-5L Mobility and Usual Activities than other patients before surgery, which may reflect (clinician or self-) selection^28^. Similarly, the present study clarified the sizes of the short- and longer-term impacts of ESG and OSR, including the prolonged effect on four domains of HRQoL for OSR, albeit small. The exception was Anxiety/Depression, which improved significantly after OSR. This is important, as many patients diagnosed with CTAA experience high levels of anxiety, due to fears that the aneurysm may rupture^4^. Provided it is safe, surgery may be a good option for individuals who find such anxiety particularly limiting.

The ETTAA study was a prospective, multicentre, longitudinal study including a relatively large number of CTAA patients and prospectively collected baseline variables. Sophisticated longitudinal modelling was used to assess changes over time and in response to intervention. There was substantial assessment of sensitivity of results to model assumptions using mixed effects ordinal logistic models, marginal models/generalized estimating equations, and bootstrapping, with very similar results, thereby increasing confidence in their validity. Missing data were also addressed using multiple imputation and joint survival-HRQoL models, with consistent results.

Despite the ordinal measurement scales for the EQ-5D-5L questionnaire domains, linear models were chosen for analysis, because they allow easier interpretation of estimates and so that analogous joint models for HRQoL and survival could be fitted. This sometimes resulted in poorly fitting models for heavily skewed data, such as Self-Care. However, given the large sample, estimates are likely to be robust. Further reassurance was provided by the extensive sensitivity analysis, including proportional odds models, which gave qualitatively similar results.

Because this was an observational study, there was no attempt to interfere with clinical practice. Thus, it is not possible to assert that associations are causal or rule out confounders. Selection for surgery is almost certainly related to factors that are impossible to measure and adjust for^28^.

Without surgical intervention, HRQoL declines slowly, but significantly, as age increases. HRQoL is impaired in the first 6 weeks after ESG and OSR, but largely returns to pre-intervention levels thereafter, taking longer after OSR due to the invasive nature, longer hospital stay, and associated complications. Otherwise, impaired HRQoL is most associated with increasing NYHA class, use of health/social care, older age, and female sex. Smoking is the only potentially reversible activity that is associated with poor HRQoL.

## Supplementary Material

znae228_Supplementary_Data

## Data Availability

The data underlying this article cannot be shared publicly to maintain the privacy of the individuals that participated in the study. The data will be shared on reasonable request to the senior author (S.R.L.; s.large@nhs.net).
