# Supplementary material for: Longitudinal health-related quality of life in people with thoracic aortic aneurysms
Source: Br J Surg. 2024 Sep 11;111(9):znae228. doi: 10.1093/bjs/znae228 (PMC11387963; doi:10.1093/bjs/znae228)
Supplement: znae228_Supplementary_Data [file znae228_supplementary_data.docx]

# **Longitudinal health-related quality of life in people with thoracic aortic aneurysms**

Authors: Linda D Sharples, Vasiliki Anagnostopoulou, Anna L Pouncey, Carol Freeman, Andrew McCarthy, Joanne Gray, Peter McMeekin, Priya Sastry, Luke Vale, Colin Bicknell, Stephen R Large for the ETTAA study investigators

Supplementary information

Contents

[Statistical analysis 3](#_Toc172096372)

[Methods 3](#_Toc172096373)

[Final models 4](#_Toc172096374)

[Baseline characteristics 5](#_Toc172096375)

[ST1 Summaries of baseline and clinical characteristics by final management group 5](#_Toc172096376)

[ST2 Summaries of comorbidities and medication use at baseline by final management group 7](#_Toc172096377)

[ST3 Summary of mortality, post-operative hospital stay and complications for ESG and OSR patients. 9](#_Toc172096378)

[ST4 Counts (%) of baseline scores of the EQ-5D-5L domains by final management group. 11](#_Toc172096379)

[Final models using complete cases. 16](#_Toc172096380)

[ST5 Final models for Mobility using complete cases. 16](#_Toc172096381)

[ST6 Final model for Self-Care using complete cases. 17](#_Toc172096382)

[ST7 Final model for Usual Activities using complete cases. 18](#_Toc172096383)

[ST8 Final model for Pain/Discomfort using complete cases. 19](#_Toc172096384)

[ST9 Final model for Anxiety/Depression using complete cases. 20](#_Toc172096385)

[Sensitivity of results due to missing data 21](#_Toc172096386)

[Initial analysis 21](#_Toc172096387)

[Multiple Imputation 21](#_Toc172096388)

[ST10 Final model for Mobility using combined estimates from 15 imputed datasets. 23](#_Toc172096389)

[Joint models for survival and HRQoL 24](#_Toc172096390)

[Methods 24](#_Toc172096391)

[ST11 Final joint model for survival and Mobility using complete data. 25](#_Toc172096392)

[ST12 Final linear mixed model for Mobility, adjusted to be comparable to the joint survival-Mobility model 26](#_Toc172096393)

[ST13 Coefficients for association between group and one unit increase in pre-operative HRQoL and the outcome post-operative HRQoL (linear regression). 27](#_Toc172096394)

[ST14 Odds ratios for the association between one unit increase in pre-operative HRQoL and incidence of any post-operative complication in ESG and OSR patients separately (logistic regression). 27](#_Toc172096395)

## Statistical analysis

### Methods

Because data were longitudinal and questionnaires were not always completed on the scheduled date, repeated measurements were analysed using mixed models with continuous time, using the actual date of completion rather than the nominal time point.

We used linear mixed effects models, in which we assume that the five levels of each domain can be seen as each representing a latent continuous variable which describes a participant’s HRQoL. Average effects are then asymptotically unbiased and allow easy interpretability of the coefficients.

Model fitting was in two parts. First, the fixed effects part of each model was ‘saturated’ by including all potential explanatory variables, time-squared, and all possible two-way interactions, with an unstructured correlation matrix using Restricted Maximum Likelihood (REML). The following variables were included:

- baseline continuous variables (all centred): age, height, BMI, diameter of the largest aneurysm,
- binary variables: Sex (male, female), location of maximum aneurysm (ascending/arch aorta, descending/thoraco-abdominal aorta), the need for additional care (formal/ informal care, no care), comorbidities (extracardiac arteriopathy, heart valve disease, connective tissue disorder, coronary artery disease, chronic obstructive pulmonary disease), treatment with statins,
- multi-level factors: smoking history (non-smoker, ex-smoker, current smoker), and New York Heart Association Classification (I, II, II, and IV).
- We also explored an 8-level variable for treatment group/period (CM, ESG pre-intervention, ESG within 6 weeks, ESG >6 weeks post-intervention, OSR pre-intervention, OSR within 6 weeks, OSR >6 weeks after intervention, WW).

Using this ‘saturated’ model, there was strong evidence for both random intercept and slope for participants, which captured unexplained variability in HRQoL between individuals at recruitment and over time. There was also evidence that increasing NYHA class was associated with increasing residual standard deviation, and this was incorporated into the models (Level 1 heterogeneity). Once the random components were decided, Wald tests, Akaike information Criteria (AIC) / Bayesian Information Criteria (BIC), were used to simplify the fixed effects structure. In some cases, interactions were removed to allow more parsimonious models, using AIC/BIC tests, after checking that the main coefficients of interest (time and interventions) were unaffected.

Models were assessed using plots of level 1 and 2 residuals and by plotting observed against model predicted trajectories. Outliers were also explored to understand the causes and potential consequences for the models.

### Final models

The final fitted models for all five domains are provided below. Main results are reported in the paper. Random effects measure variation between participants at baseline (intercept) and in changes over time (slope) that is not explained by variables in the model. All domains of HRQoL exhibited significant unexplained participant variation at baseline and in changes over time. For Anxiety/Depression there was a negative correlation between these two components, suggesting that participants with worse Anxiety/Depression at baseline had slower additional increase in this domain. For the other four domains of HRQoL, correlation between random effects at baseline and in slope was small and not significant.

## Baseline characteristics

### ST1 Summaries of baseline and clinical characteristics by final management group

|  | All  (n=886) | WW  (n=489) | CM  (n=112) | ESG  (n=150) | OSR  (n=135) | p-value |
| --- | --- | --- | --- | --- | --- | --- |
| Age (years) |  |  |  |  |  | <0.0001 |
| Mean (SD)  Range | 70.8 (10.9)  26, 92 | 70.8 (10.7)  32, 92 | 76.6 (9.9)  26, 92 | 72.0 (8.6)  49, 89 | 64.9 (11.6)  31, 83 |  |
| Sex,n (%) |  |  |  |  |  | 0.430 |
| Female  Male | 321 (36.2)  565 (63.8) | 174 (35.6)  315 (64.4) | 48 (42.9)  64 (57.1) | 50 (33.3)  100 (66.7) | 49 (36.3)  86 (63.7) |  |
| Height (cm) |  |  |  |  |  | 0.0001 |
| Mean(SD)  Missing (%) | 171.0 (10.8)  35 (4.0) | 171.3 (10.2)  19 (3.9) | 167.4 (12.6)  9 (8.0) | 169.9 (10.2)  4 (2.7) | 173.7 (11.3)  3 (2.2) |  |
| Weight (kg) |  |  |  |  |  | 0.0001 |
| Mean(SD)  Missing (%) | 80.0 (17.2)  38 (4.3) | 80.6 (17.2)  22 (4.9) | 74.2 (17.3)  8 (7.1) | 78.6 (15.5)  5 (3.3) | 83.9 (17.5)  3 (2.2) |  |
| BMI (kg/m2) |  |  |  |  |  | 0.1885 |
| Mean(SD)  Missing (%) | 27.3 (4.8)  42 (4.7) | 27.5 (5.0)  24 (4.9) | 26.5 (4.9)  9 (8.0) | 27.1 (4.3)  6 (4.0) | 27.7 (4.6)  3 (2.2) |  |
| Smoking, n (%) |  |  |  |  |  | 0.3530 |
| Never  Ex  Current  Missing | 263 (29.7)  503 (56.8)  113 (12.7)  7 (0.8) | 142 (29.0)  284 (58.1)  59 (12.1)  4 (0.8) | 40 (35.7)  57 (50.9)  14 (12.5)  1 (0.9) | 36 (24.0)  93 (62.0)  20 (13.3)  1 (0.7) | 45 (33.3)  69 (51.1)  20 (14.8)  1 (0.7) |  |
| Care, n (%) |  |  |  |  |  | 0.002 |
| None  Formal/Informal  Missing | 776 (87.6)  103 (11.6  7 (0.8) | 425 (86.9)  60 (12.2)  4 (0.8) | 88 (78.6)  23 (20.5)  1 (0.9) | 138 (92.0)  12 (8.0)  0 (0.0) | 125 (92.6)  8 (5.9)  2 (1.5) |  |
| Max diameter (cm) |  |  |  |  |  | <0.0001 |
| Mean(SD) | 5.7 (1.1) | 5.3 (1.0) | 6.3 (1.2) | 6.0 (1.1) | 6.3 (1.0) |  |
| Max. site, n (%) |  |  |  |  |  | <0.001 |
| Arch/Ascending  Descending/Thoraco-abdominal | 152 (17.2)  734 (82.8) | 87 (17.8)  402 (82.2) | 22 (19.6)  90 (80.4) | 2 (1.3)  148 (98.7) | 41 (30.4)  94 (69.6) |  |

### ST2 Summaries of comorbidities and medication use at baseline by final management group

|  | All  (n=886) | WW  (n=489) | CM  (n=112) | ESG  (n=150) | OSR  (n=135) | p-value |
| --- | --- | --- | --- | --- | --- | --- |
| Connective tissue disorder, n (%) | |  |  |  |  | <0.0001 |
| Yes  No | 55 (6.2)  831 (93.8) | 30 (6.1)  459 (93.9) | 3 (2.7)  109 (97.3) | 2 (1.3)  148 (98.7) | 20 (14.8)  115 (85.2) |  |
| COPD, n (%) |  |  |  |  |  | 0.430 |
| Yes  No  Missing | 163 (18.4)  718 (81.0)  5 (0.6) | 87 (17.8)  397 (81.2)  5 (1.0) | 26 (23.2)  86 (76.8)  0 (0.0) | 32 (21.3)  118 (78.7)  0 (0.0) | 18 (13.3)  117 (86.7)  0 (0.0) |  |
| Coronary artery disease, n (%) | |  |  |  |  | 0.0001 |
| Yes  No  Missing | 198 (19.0)  701 (79.1)  17 (1.9) | 99 (20.2)  377 (77.1)  13 (2.7) | 25 (22.3)  85 (75.9)  2 (1.79) | 26 (17.3)  123 (82.0)  1 (0.74) | 18 (13.3)  116 (85.9)  1 (0.7) |  |
| Diabetes, n (%) |  |  |  |  |  | 0.0001 |
| Type I/II  None  Missing | 83 (9.4)  800 (90.3)  3 (0.3) | 54 (11.0)  432 (88.3)  3 (0.6) | 7 (6.3)  105 (93.7)  0 (0.0) | 13 (8.7)  137 (91.3)  0 (0.0) | 9 (6.7)  126 (93.3)  0 (0.0) |  |
| Extracardiac arteriopathy, n (%) | |  |  |  |  | 0.1885 |
| Yes  No  Missing | 133 (15.0)  738 (83.3)  15 (1.7) | 71 (14.5)  406 (83.0)  12 (2.5) | 20 (17.9)  91 (81.2)  1 (0.9) | 26 (17.3)  123 (82.0)  1 (0.7) | 16 (11.9)  118 (87.4)  1 (0.7) |  |
| Hypertension, n (%) |  |  |  |  |  | 0.3530 |
| Yes  No  Missing | 775 (87.7)  109 (12.3)  2 (0.2) | 424 (87.1)  63 (13.0)  2 (0.4) | 97 (86.6)  15 (13.4)  0 (0.0) | 135 (90.0)  15 (10.0)  0 (0.0) | 119 (88.2)  16 (11.8)  0 (0.0) |  |
| NYHA class, n (%) |  |  |  |  |  | 0.002 |
| I  II  III  IV  Missing | 359 (40.5)  315 (35.6)  150 (16.9)  26 (2.9)  36 (4.1) | 198 (40.5)  175 (35.8)  86 (17.6)  16 (3.3)  14 (2.9) | 39 (34.8)  41 (36.6)  27 (24.1)  3 (2.7)  2 (1.8) | 68 (45.3)  47 (31.3)  20 (13.3)  4 (2.7)  11 (7.3) | 54 (40.0)  52 (38.5)  17 (12.6)  3 (2.2)  9 (6.7) |  |
| Valvular heart disease, n (%) | |  |  |  |  | <0.0001 |
| Yes  No  Missing | 165 (18.6)  706 (79.7)  15 (1.7) | 89 (18.2)  389 (79.6)  11 (2.2) | 23 (20.5)  87 (77.7)  2 (1.8) | 15 (10.0)  134 (89.3)  1 (0.7) | 38 (28.2)  96 (71.1)  1 (0.7) |  |
| Statins, n (%) |  |  |  |  |  | <0.001 |
| Yes  No  Missing | 512 (57.8)  372 (42.0)  2 (0.4) | 283 (57.9)  204 (41.7)  0 (0.0) | 72 (64.3)  40 (35.7)  0 (0.0) | 106 (70.7)  44 (29.3)  0 (0.0) | 51 (37.8)  84 (62.2)  0 (0.0) |  |

### ST3 Summary of mortality, post-operative hospital stay and complications for ESG and OSR patients.

| Outcome | ESG (n=150) | OSR (n=135) |
| --- | --- | --- |
| ICU stay (days) |  |  |
| Median (Quartiles)  Missing n | 0.5 (0, 3)  2 | 5 (3, 10)  2 |
| Total length of stay (days) |  |  |
| Median (Quartiles)  Missing n | 7 (4, 12)  3 | 16 (10, 23)  4 |
| Operative outcome n (%)  Discharged alive  Aneurysm-related death  Other cause death | 142 (94.7%)  5 (3.3%)  3 (2.0%) | 120 (88.9%)  11 (8.1%)  4 (3.0%) |
| Complications |  |  |
| Myocardial infarction n (%)  Missing n | 9 (6.1%)  2 | 2 (1.5%)  0 |
| Cardiac support n (%)  Intra-aortic Balloon Pump  Inotropes  Missing n | 0 (-)  27 (18.5%)  3 | 1 (0.8%)  78 (58.7%)  2 |
| Prolonged ventilation > 48 hours n (%)  Missing n | 5 (3.4%)  4 | 37 (28.0%)  3 |
| Renal support n (%)  Missing n | 2 (1.4%)  3 | 15* (11.3%)  2 |
| Gastro-intestinal n (%)  Bleeding  Ischaemia  Other  Missing n | 3 (2.0%)  2 (1.4%)  2 (1.4%)  2 | 3 (2.2%)  2 (1.5%)  7 (5.2%)  0 |
| Neurological injury n (%)  Cerebrovascular accident  Transient Ischaemic attack  Missing n | 4 (2.7%)  1 (0.7%)  2 | 11 (8.2%)  2 (1.5%)  1 |
| Spinal cord injury n (%)  Paraparesis  Paraplegia  Missing n | 2 (1.4%)  3 (2.0%)  2 | 0 (-)  4 (3.0%)  0 |
| Vocal cord palsy n (%)  Missing n | 2 (1.4%)  8 | 7 (5.9%)  17 |
| Thromboembolic event n (%)  Deep Vein Thrombosis  Pulmonary embolism  Type not recorded  Missing n | 0 (-)  1 (0.7%)  1 (0.7%)  2 | 3 (2.2%)  3 (2.2%)  1 (0.8%)  0 |
| Infection n (%)  Wound  Prosthesis  Other  Missing n | 2 (1.4%)  1 (0.7%)  14 (9.5%)  2 | 4 (3.0%)  1 (0.8%)  39 (29.1%)  1 |
| Return to theatre n (%)  Missing n | 16 (10.7%)  1 | 20 (14.8%)  0 |
| Any complication n (%) | 58 (38.7%) | 103 (76.3) |
| Number per patient n-n | 1-6 | 1-8 |

*14 temporary support duration <1 to 26 days during surgical admission, one permanent support (discharged on renal support at 26 days).

Ratio of risk of at least one complication for OSR relative to ESG, adjusted for age at procedure and sex = 2.08 (1.43, 3.04).

Ratio of complication rates for OSR relative to ESG, adjusted for age at procedure and sex = 2.90 (2.07, 4.05).

### ST4 Counts (%) of baseline scores of the EQ-5D-5L domains by final management group.

| Domain | Scores | WW (n=489) | CM (n=112) | ESG (n=150) | OSR (n=135) |
| --- | --- | --- | --- | --- | --- |
| Mobility | 1  2  3  4  5  Missing | 230 (47.0%)  99 (20.2%)  105 (21.5%)  48 (9.8%)  4 (0.8%)  3 (0.6%) | 36 (32.1%)  21 (18.8%)  37 (33.0%)  15 (13.4%)  3 (2.7%)  0 (0%) | 80 (53.3%)  26 (17.3%)  25 (16.7%)  12 (8.0%)  1 (0.7%)  6 (4.0%) | 77 (57.0%)  25 (18.5%)  24 (17.8%)  7 (5.2%)  2 (1.5%)  0 (0%) |
| Self-care | 1  2  3  4  5  Missing | 396 (81.0%)  44 (9.0%)  36 (7.4%)  5 (1.0%)  5 (1.0%)  3 (0.6%) | 81 (72.3%)  20 (17.9%)  8 (7.1%)  3 (2.7%)  0 (0%)  0 (0%) | 117 (78.0%)  13 (8.7%)  9 (6.0%)  2 (1.3%)  2 (1.3%)  7 (4.7%) | 118 (87.4%)  12 (8.9%)  4 (3.0%)  1 (0.7%)  0 (0.0%)  0 (0.0%) |
| Usual activities | 1  2  3  4  5  Missing | 238 (48.7%)  108 (22.1%)  82 (16.8%)  42 (8.6%)  16 (3.3%)  3 (0.6%) | 42 (37.5%)  24 (21.4%)  28 (25.0%)  8 (7.1%)  10 (8.9%)  0 (0%) | 84 (56.0%)  26 (17.3%)  16 (10.7%)  9 (6.0%)  9 (6.0%)  6 (4.0%) | 64 (47.4%)  34 (25.2%)  24 (17.8%)  4 (3.0%)  9 (6.7%)  0 (0%) |
| Pain and discomfort | 1  2  3  4  5  Missing | 181 (37.0%)  152 (31.1%)  113 (23.1%)  38 (8.6%)  1 (0.2%)  3 (0.6%) | 47 (42%)  30 (26.8%)  25 (22.3%)  8 (7.1%)  2 (1.8%)  0 (0%) | 65 (43.3%)  43 (28.7%)  25 (16.7%)  10 (6.7%)  0 (0.0%)  7 (4.7%) | 64 (47.4%)  39 (28.9%)  21 (15.6%)  9 (6.7%)  2 (1.5%)  0 (0%) |
| Anxiety and depression | 1  2  3  4  5  Missing | 301 (61.6%)  107 (21.9%)  57 (11.7%)  15 (3.1%)  4 (0.2%)  5 (1.0%) | 65 (58.0%)  24 (21.4%)  21 (18.8%)  1 (0.9%)  1 (0.9%)  0 (0%) | 74 (49.3%)  35 (23.3%)  25 (16.7%)  7 (4.7%)  2 (1.3%)  7 (4.7%) | 68 (50.4%)  36 (26.7%)  22 (16.3%)  4 (3.0%)  5 (3.7%)  0 (0%) |

**EQ-5D-5L Mobility at baseline by final management group**


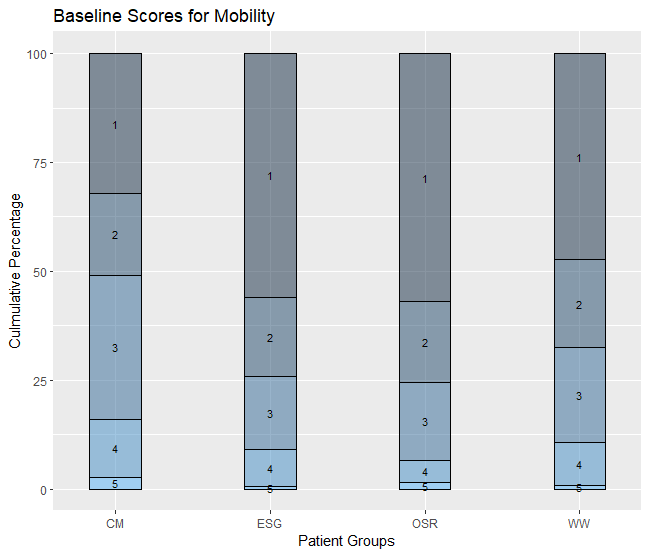


**EQ-5D-5L Self-Care at baseline by final management group**


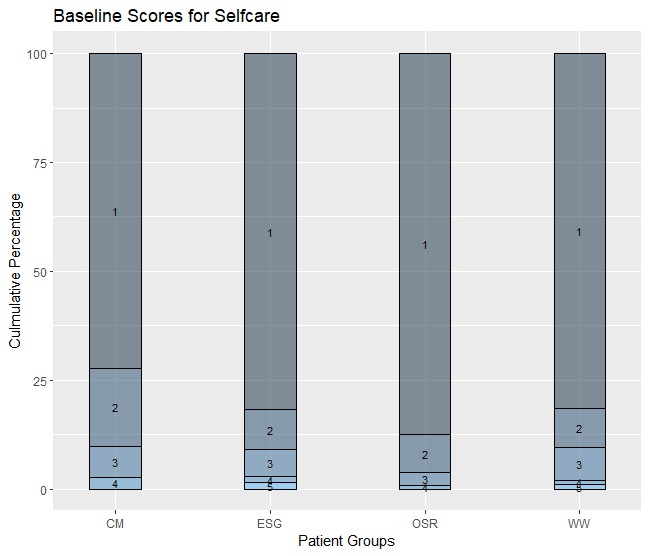


**EQ-5D-5L Usual Activities at baseline by final management group**


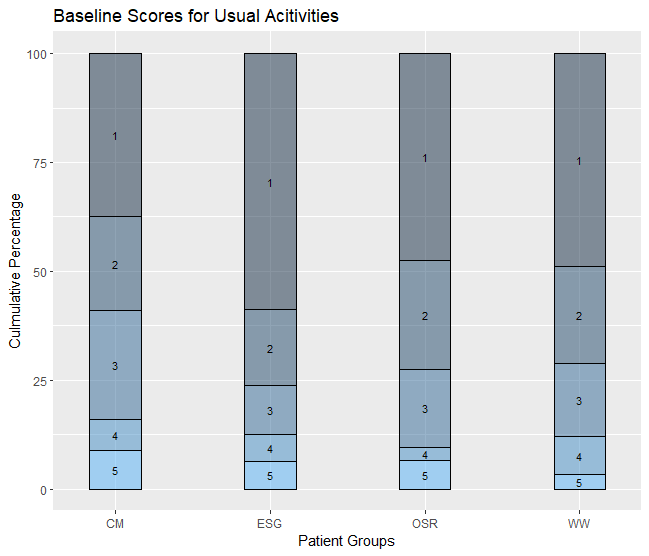


**EQ-5D-5L Pain/Discomfort at baseline by final management group**


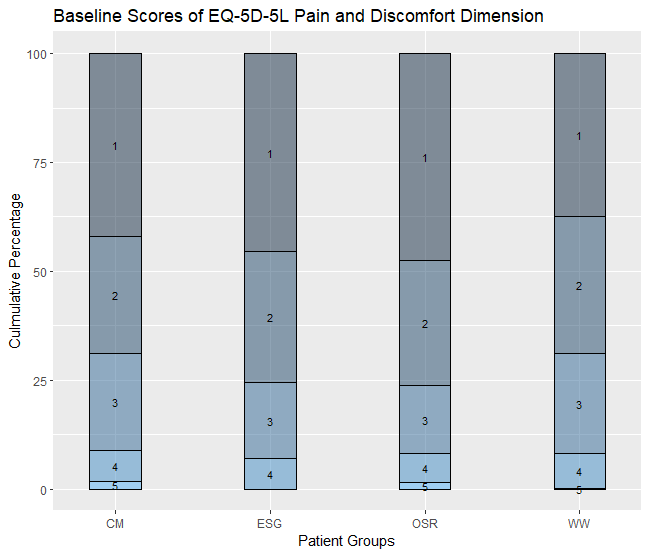


**EQ-5D-5L Anxiety/Depression at baseline by final management group**


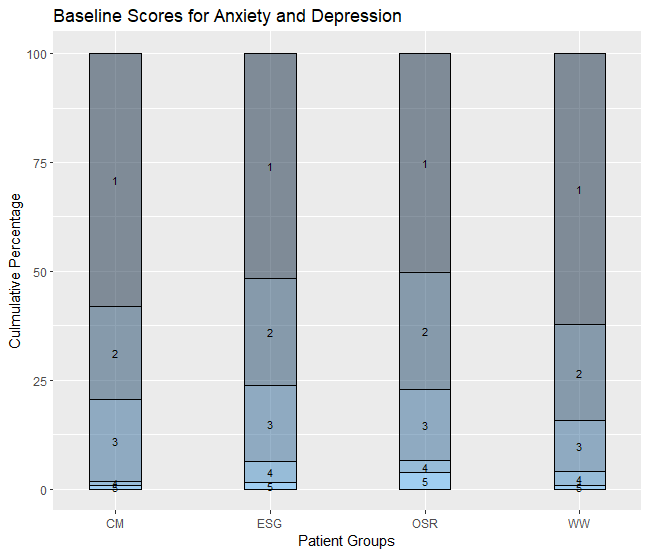


## Final models using complete cases.

### ST5 Final models for Mobility using complete cases.

### ST6 Final model for Self-Care using complete cases.

ST7 Final model for Usual Activities using complete cases.****

### ST8 Final model for Pain/Discomfort using complete cases.

### ST9 Final model for Anxiety/Depression using complete cases.

## Sensitivity of results due to missing data

### Initial analysis

Missing data occurred due to incomplete baseline characteristics and non-completion of forms.

For the 886 participants, the number of missing baseline characteristics were 35 (3.95%) for height, 38 (4.29%) for weight, 42 (4.74%) for BMI, 15 (1.69%) for extracardiac arteriopathy, 15 (1.69%) for heart valve disease, 7 (0.79% ) for smoking status, 5 (0.56%) for COPD, 36 (4.06%) for New York Heart Association dyspnoea class, 7 (0.79%) for care status, 17 (1.9%) for coronary artery disease, 3 (0.3%) for diabetes status, 2 (0.2%) for hypertension and 2 (0.2%) for use of statins. Recording of creatinine, haemoglobin and presence of left ventricular ejection fraction were not mandated and were either missing or not measured for more than 45% of participants. Also, they were more likely to be collected if the participant was being considered for a procedure in the near future (missing not at random). Thus, they were not used in modelling or included in descriptive statistics. Excluding variables which were not measured in all sites, 777 participants (87.7%) had complete baseline characteristics (4091 (89.5%) questionnaires), and 109 (12.3%) had at least one baseline characteristic missing (479 (10.5%) questionnaires). Using a series of logistic regression models, we found little evidence that HRQoL was associated with missing baseline characteristics overall.

Non-completion of forms was due to staggered entry of participants to ETTAA, participant withdrawal especially after the minimum follow up period of 12 months, resetting of follow-up times after interventions and death. Overall, exploration found little to no evidence against the assumption that data were “missing completely at random”, hence the decision to use a complete-case analysis. This assumes that data are “missing at random” conditional on observed data.

Sensitivity of results to missing data was assessed by (i) using multiple imputation and (ii) fitting joint models of survival and HRQoL for each domain separately, to adjust for those with the worst HRQoL leaving the study early.

### Multiple Imputation

We used multiple imputation using chained equations (MICE) to create 15 complete data sets. Predictive mean matching was used for covariates. Variables in the imputation model were, care, smoking, NYHA, COPD sex, age, diameter of maximum aneurysm, site of maximum aneurysm, final survival status, connective tissue disorder, as well as time (in years) and the five outcome variables. All linear mixed effects models were refitted using for the 15 complete datasets, with fixed effects combined using Rubin’s rules. Estimates were almost identical to the estimates from the complete-case analysis. Below, we provide results for Mobility as an example.

### ST10 Final model for Mobility using combined estimates from 15 imputed datasets.

We note that there are only minor differences from the complete case analysis for both point estimates and 95% confidence intervals. This was true of all five domains and results can be provided on request.

## Joint models for survival and HRQoL

### Methods

Because the longitudinal trajectories may be affected by informative drop-out, joint models of the longitudinal data and survival were fitted using the *stjm* command in Stata 18 using the stjm command. A Weibull model was used for survival because the Cox model has not yet been implemented in this software. The survival model included age at recruitment, sex, diameter of largest aneurysm and NYHA class as a continuous variable. The longitudinal HRQoL data was fitted using an unstructured covariance matrix, but the software does not yet allow for level 1 heterogeneity of residuals. The interaction between the first 6 weeks post-procedure and sex from the Usual Activities model was also not possible in this software. Otherwise, the structures of the models were identical to the primary analysis. These models were compared with linear mixed models with the adjustments required for the joint model (e.g. no level 1 heterogeneity, NYHA as a continuous variable).

Results for the joint model for Mobility are given below as an example, as well as the analogous linear mixed model. We note that these estimates are very similar, suggesting that dropout due to death had minimal effect on the estimates of average Mobility over time.

### ST11 Final joint model for survival and Mobility using complete data.

### ST12 Final linear mixed model for Mobility, adjusted to be comparable to the joint survival-Mobility model

### ST13 Coefficients for association between group and one unit increase in pre-operative HRQoL and the outcome post-operative HRQoL (linear regression).

| HRQoL domain | Coefficient for pre-operative HRQoL (95% CI) | Coefficient for OSR relative to ESG (95%CI) |
| --- | --- | --- |
| Mobility | 0.55 (0.43, 0.67) | 0.25 (0.00, 0.50) |
| Self Care | 0.50 (0.34, 0.65) | 0.25 (0.03, 0.46) |
| Usual Activities | 0.40 (0.26, 0.54) | 0.77 (0.45, 1.10) |
| Pain/Discomfort | 0.45 (0.31, 0.59) | 0.18 (-0.08, 0.44) |
| Anxiety/Depression | 0.37 (0.26, 0.47) | -0.03 (-0.25, 0.19) |

### ST14 Odds ratios for the association between one unit increase in pre-operative HRQoL and incidence of any post-operative complication in ESG and OSR patients separately (logistic regression).

| HRQoL domain | Odds ratio per unit for ESG (95% CI) | Odds ratio per unit for OSR (95%CI) |
| --- | --- | --- |
| Mobility | 0.92 (0.71, 1.20) | 1.85 (1.24, 2.76) |
| Self Care | 1.06 (0.71, 1.60) | 3.16 (1.66, 6.03) |
| Usual Activities | 0.87 (0.68, 1.11) | 1.59 (1.17, 2.15) |
| Pain/Discomfort | 0.99 (0.74, 1.32) | 1.77 (1.24, 2.52) |
| Anxiety/Depression | 0.96 (0.72, 1.27) | 1.92 (1.34, 2.74) |
